# Serum Neurofilament Light and GFAP Are Associated With Disease Severity in Inflammatory Disorders With Aquaporin-4 or Myelin Oligodendrocyte Glycoprotein Antibodies

**DOI:** 10.3389/fimmu.2021.647618

**Published:** 2021-03-16

**Authors:** Xuechun Chang, Wenjuan Huang, Liang Wang, Jingzi ZhangBao, Lei Zhou, Chuanzhen Lu, Min Wang, Jian Yu, Haiqing Li, Yuxin Li, Chongbo Zhao, Jiahong Lu, Chao Quan

**Affiliations:** ^1^Department of Neurology, Huashan Hospital, Shanghai Medical College, Fudan University, Shanghai, China; ^2^Department of Ophthalmology and Vision Science, Eye and ENT Hospital, Shanghai Medical College, Fudan University, Shanghai, China; ^3^Department of Radiology, Huashan Hospital, Shanghai Medical College, Fudan University, Shanghai, China

**Keywords:** neurofilament light, glial fibrillary acidic protein, aquaporin-4, myelin oligodendrocyte glycoprotein, neuromyelitis optica spectrum disorder

## Abstract

**Objective:** To evaluate the potential of serum neurofilament light (sNfL) and serum glial fibrillary acidic protein (sGFAP) as disease biomarkers in neuromyelitis optica spectrum disorder (NMOSD) with aquaporin-4 antibody (AQP4-ab) or myelin oligodendrocyte glycoprotein-antibody-associated disease (MOGAD).

**Methods:** Patients with AQP4-ab-positive NMOSD (*n* = 51), MOGAD (*n* = 42), and relapsing-remitting multiple sclerosis (RRMS) (*n* = 31 for sNfL and *n* = 22 for sGFAP testing), as well as healthy controls (HCs) (*n* = 28), were enrolled prospectively. We assessed sNfL and sGFAP levels using ultrasensitive single-molecule array assays. Correlations of sNfL and sGFAP levels with clinical parameters were further examined in AQP4-ab-positive NMOSD and MOGAD patients.

**Results:** sNfL levels were significantly higher in patients with AQP4-ab-positive NMOSD (median 17.6 pg/mL), MOGAD (27.2 pg/mL), and RRMS (24.5 pg/mL) than in HCs (7.4 pg/mL, all *p* < 0.001). sGFAP levels were remarkably increased in patients with AQP4-ab-positive NMOSD (274.1 pg/mL) and MOGAD (136.7 pg/mL) than in HCs (61.4 pg/mL, both *p* < 0.001). Besides, sGFAP levels were also significantly higher in patients with AQP4-ab-positive NMOSD compared to those in RRMS patients (66.5 pg/mL, *p* < 0.001). The sGFAP/sNfL ratio exhibited good discrimination among the three disease groups. sNfL levels increased during relapse in patients with MOGAD (*p* = 0.049) and RRMS (*p* < 0.001), while sGFAP levels increased during relapse in all three of the disease groups (all *p* < 0.05). Both sNfL and sGFAP concentrations correlated positively with Expanded Disability Status Scale scores in AQP4-ab-positive NMOSD (β = 1.88, *p* = 0.018 and β = 2.04, *p* = 0.032) and MOGAD patients (β = 1.98, *p* = 0.013 and β = 1.52, *p* = 0.008).

**Conclusion:** sNfL and sGFAP levels are associated with disease severity in AQP4-ab-positive NMOSD and MOGAD patients, and the sGFAP/sNfL ratio may reflect distinct disease pathogenesis.

## Introduction

Neuromyelitis optica spectrum disorder (NMOSD) comprises a spectrum of inflammatory autoimmune disorders of the central nervous system (CNS) with a predilection for the optic nerves and spinal cord (1). The majority of NMOSD patients have antibodies to aquaporin-4 (AQP4) water channels, which are situated predominantly on the end-feet of astrocytic processes (2, 3). Therefore, AQP4-antibody-positive NMOSD (AQP4-ab+NMOSD) is considered to be an autoimmune astrocytopathy with secondary demyelination. Using cell-based assays, myelin oligodendrocyte glycoprotein antibodies (MOG-ab) have been detected in a subset of NMOSD patients that are AQP4-ab negative (4, 5). However, neuropathologic findings of cases with MOG-ab show predominant demyelination yet preservation of astrocytes, distinct from the astrocytopathy observed in AQP4-ab+NMOSD (6, 7). Hence, MOG-ab-associated disease (MOGAD) has recently been proposed as a distinct disease entity independent of NMOSD (8, 9).

NMOSD is potentially life-threatening; unpredictable relapses result in cumulative neurological disabilities. Some patients with MOGAD are also severely disabled, and the majority of MOGAD patients experience relapses. However, serum biomarker investigations for these two diseases are still lacking.

Neurofilament light chain (NfL) is a component of the neuronal cytoskeleton and is released into the cerebrospinal fluid (CSF) and blood after neuronal-axonal injury (10, 11). Serum NfL (sNfL) measured via ultra-sensitive single-molecule arrays (SIMOA) has been observed to be increased in multiple neurological diseases—including neurodegenerative disorders, trauma, and multiple sclerosis (MS)—and strongly correlates with CSF NfL (12–15). sNfL is currently recognized as a feasible biomarker that reflects MS disease severity, treatment efficacies, and prognosis (15–20). Glial fibrillary acidic protein (GFAP) is a principal intermediate filament that contributes to the astrocytic cytoskeleton and represents a marker of astrocytic injury (21–24). Previous reports have also shown increased CSF and serum GFAP (sGFAP) in MS, especially at progressive stages (25, 26).

Recently, sNfL levels have been shown to be increased significantly in patients with MOGAD compared with those of controls (27, 28), and correlate with disease severity (28, 29). Moreover, elevated GFAP and NfL levels in the serum or CSF of patients with AQP4-ab+NMOSD have been reported (27, 30, 31), especially in the acute relapse phase (29, 30), and are associated with increased Expanded Disability Status Scale (EDSS) scores (29, 30). Notably, investigators have proposed that a higher sGFAP/sNfL ratio at relapse differentiates NMOSD from MS, reflecting distinct disease mechanisms (30).

In the current study, we compared sNfL and sGFAP levels, as well as sGFAP/sNfL ratios, among Chinese healthy controls (HCs) and patients with AQP4-ab+NMOSD, MOGAD, and MS. Furthermore, we examined the clinical relevance of sNfL and sGFAP in AQP4-ab+NMOSD and MOGAD.

## Materials and Methods

### Participants

This was a single-center prospective sample collection study. Serum samples from 51 patients with AQP4-ab+NMOSD (51 samples), 42 patients with MOGAD (42 samples), and 31 patients with relapsing-remitting MS (RRMS; for sNfL, 31 samples from 31 patients; for sGFAP, 22 samples from 22 patients) were collected from June 2018 to December 2019 at NMO-MS Clinic of Huashan Hospital in Shanghai, China.

Diagnosis of AQP4-ab+NMOSD was based on the criteria established by the International Panel in 2015 (32). MOGAD was defined as CNS demyelinating syndromes associated with positive serum MOG-antibodies (8, 33), and diagnostic consensus criteria including red flags were checked to enhance the diagnosis certainty for MOGAD (33). Only patients with an AQP4-ab or MOG-ab titer ≥ 1:32 were included. RRMS was diagnosed according to the 2010 McDonald criteria (34). An attack or relapse was defined according to the descriptions in the 2010 McDonald criteria. Disability was evaluated via EDSS scores. The demographic and clinical data of the patients were recorded.

Blood specimens were drawn from the cubital vein, centrifuged, and stored at −80°C until the testing. Samples drawn “during relapse” denote that they were taken within 60 days after the onset of a recent relapse. Samples drawn “during remission” denote that the blood draw occurred more than 60 days after a recent relapse. Serum samples (*n* = 28) were also obtained from 28 HCs that were age-matched to the three disease groups.

### Standard Protocol Approval, Registrations, and Patient Consents

This study was approved by the Medical Ethics Committee of Huashan Hospital affiliated to Fudan University and was therefore performed in accordance with the ethical standards established in the 1964 Declaration of Helsinki and its later amendments. Written informed consent was obtained from each participant prior to his/her inclusion in the study.

### Antibody Detection

Anti-MOG-immunoglobulin G (IgG) and anti-AQP4-IgG were determined using a fixed cell-based indirect immune-fluorescence test (Euroimmun AG, Lübeck, Germany) and all the testing results were confirmed twice.

### Assessments of sNfL and sGFAP

sNfL and sGFAP concentrations were analyzed in duplicates using SIMOA. Nf-light kits and GFAP discovery kits were used with an HD-1 immunoassay analyzer (Quanterix, Boston, Massachusetts, USA). Sera were diluted (1:4), as recommended by the manufacturer, and concentrations were calculated using the corresponding standard curve. The analyses were performed by a board-certified laboratory technician, blinded to the corresponding clinical data, using one batch of reagents. The intra-assay coefficients of variations (CVs) for duplicate determinations of concentrations were within 10%, and a CV of lower than 10% was required for an analysis to be considered valid. Inter-assay coefficients of variations were within 12%. All samples produced signals above the analytic sensitivity of the corresponding assay. Seven serum samples exhibited GFAP values above the highest calibrator (> 4,000 pg/mL) and were consequently remeasured at appropriately higher dilutions. The ratio of sGFAP and sNfL was calculated for each participant as described previously (30).

### Statistical Analysis

Statistical analysis was performed via IBM SPSS 24.0 (IBM Corp., Armonk, NY, USA). Graphs were generated with GraphPad Prism 6.0 (GraphPad Software Inc., San Diego, CA, USA) and R 3.5.0 (R Foundation for Statistical Computing, Vienna, Austria). Categorical variables are described by counts and percentages. Continuous variables are described by medians and interquartile ranges (IQRs). Kruskal–Wallis tests with Dunn's multiple comparisons and Fisher's exact tests were used for comparisons of demographic data.

sGFAP and sNfL levels were log-transformed when analyzed. Kolmogorov-Smirnov test (when sample size ≥ 50) and Shaprio-Wilk test (sample size < 50) were performed for the demonstration of normal distribution. Associations of sNfL or sGFAP levels with age in HCs were assessed by linear regression models. Analysis of covariance (ANCOVA) was performed—considering log sNfL and sGFAP levels as dependent variables, groups (NMOSD, MOGAD, and RRMS) as fixed variables, and age and EDSS scores as covariates—to examine differences between sNfL and sGFAP levels among the various groups. Bonferroni (equal variance assumed) or Dunnett's T3 method (equal variance not assumed) were applied to correct for *post-hoc* multiple comparisons.

To investigate correlations between clinical parameters and sNfL/sGFAP levels, we first conducted linear regression to identify clinical variables with significant associations. Then, multiple linear-regression analyses were performed by applying identified variables, age, and EDSS scores as independent variables by the forced-entry method. Regression coefficients were back-transformed to the original scale (β). A value of *p* < 0.05 was considered statistically significant.

Correlation analyses were performed only in the whole AQP4-ab+NMOSD and MOGAD groups, and were not done in relapse/remission subgroups or in the RRMS group due to the limited sample size.

### Data Availability

Anonymized data not exhibited in our study will be made available upon request from qualified investigator.

## Results

### Demographic and Clinical Data

The demographic and clinical data of the included patients with AQP4-ab+NMOSD, MOGAD, and RRMS, as well as those of the 28 HCs are listed in Table 1. The ages at sampling, disease durations (since the onset of the first episode), and the intervals from a recent relapse to sampling were comparable across the three disease groups (Table 1). No patients were double positive for AQP4-ab and MOG-ab.

**Table 1:** Demographic and clinical data of participants at baseline.

	**AQP4 (*n* = 51)**	**MOG^**‡**^ (*n* = 42)**	**RRMS (*n* = 31)**	**HC (*n* = 28)**	***p*****-value**					
					**AQP4 vs. MOG**	**AQP4 vs. RRMS**	**MOG vs. RRMS**	**AQP4 vs. HC**	**MOG vs. HC**	**RRMS vs. HC**
Female, *n* (%)	44 (86.3)	22 (52.4)	17 (54.8)	12 (57.1)	**0.001**	**0.001**	0.835	**0.001**	0.256	0.373
Age at sampling, median (IQR), y	37 (24–48)	33 (24–41)	31 (25–38)	35 (24–47)	0.181	0.235	1.000	1.000	0.567	0.612
Age at onset, median (IQR), y	33 (20–47)	27 (17–38)	24 (22–32)	–	0.307	0.439	1.000	–	–	–
Phase (relapse/remission), n	37/14	23/19	17/14	–	–	–	–	–	–	–
Disease duration, median (IQR), m	17 (5–66)	9.5 (1–48)	17 (5–76)	–	0.946	1.000	0.992	–	–	–
Total number of attacks, median (range)	2 (1–5)	2 (1–12)	2 (1–6)	–	0.885	0.885	1.000	–	–	–
Intervals from recent relapse to sampling, median (IQR), d	24 (13–61)	48 (19–91)	50 (30–97)	–	0.220	0.193	1.000	–	–	–
Sampling during relapse	16 (10.2–30)	20 (13–27)	30 (12–40)	–	0.537	0.357	0.405	–	–	–
Sampling during remission	92 (82–180)	103 (68.7–345)	92.5 (71.8–142.5)	–	0.519	0.519	1.000	–	–	–
EDSS score at sampling, median (IQR)	3 (1.5–4)	2 (1–3)	2 (1.5–3)	–	**0.005**	0.240	1.000	–	–	–
Phenotype of a recent relapse, *n* (%)										
ON	11 (21.5)	14 (33.3)	0	–	0.647	–	–	–	–	–
Myelitis	16 (31.3)	3 (7.1)	0	–	**0.004**	–	–	–	–	–
Brain	0	10 (23.8)	5 (16.1)	–	–	–	0.561	–	–	–
ON and myelitis	8 (15.7)	3 (7.1)	0	–	0.334	–	–	–	–	–
ON and Brain	5 (9.8)	4 (9.5)	4 (12.9)	–	1.000	0.724	0.716	–	–	–
Myelitis and brain	6 (11.7)	7 (16.7)	19 (61.3)	–	0.562	**0.001**	**0.001**	–	–	–
ON, myelitis, and brain	5 (9.8)	1 (2.4)	3 (9.6)	–	0.153	1.000	0.305	–	–	–
Treatment at sampling, *n* (%)										
Steroid	24 (47.1)	29 (69.0)	8 (25.8)	–	**0.038**	0.065	**0.001**	–	–	–
Steroid and oral immunosuppressant^§^	13 (25.5)	7 (16.7)	2 (6.4)	–	0.325	**0.001**	0.286	–	–	–
Rituximab	1 (2.0)	1 (2.4)	1 (3.2)	–	0.702	1.000	1.000	–	–	–
Teriflunomide^¶^	0	0	4 (12.9)	–	–	–	–	–	–	–
None	13 (25.5)	5 (11.9)	16 (51.0)	–	0.119	**0.019**	**0.001**	–	–	–

*AQP4, aquaporin 4; MOG, myelin oligodendrocyte glycoprotein; RRMS, relapsing remitting multiple sclerosis; IQR, interquartile range; EDSS, Expanded Disability Status Scale; HC, healthy control; ON, optic neuritis; MRI, magnetic resonance imaging; y, year; d, day; m, month; AQP4 stands for AQP4-antibody-positive NMOSDs; MOG^‡^ stands for MOG-antibody-associated diseases (MOGADs); Oral immunosuppressants^§^ including azathioprine and mycophenolate mofetil; Teriflunomide^¶^ was the only disease- modifying drug for RRMS in China at the time of investigation. The p-values lower than 0.05 are in bold type*.

### sNfL and sGFAP Levels in HCs

**Figure 1:**
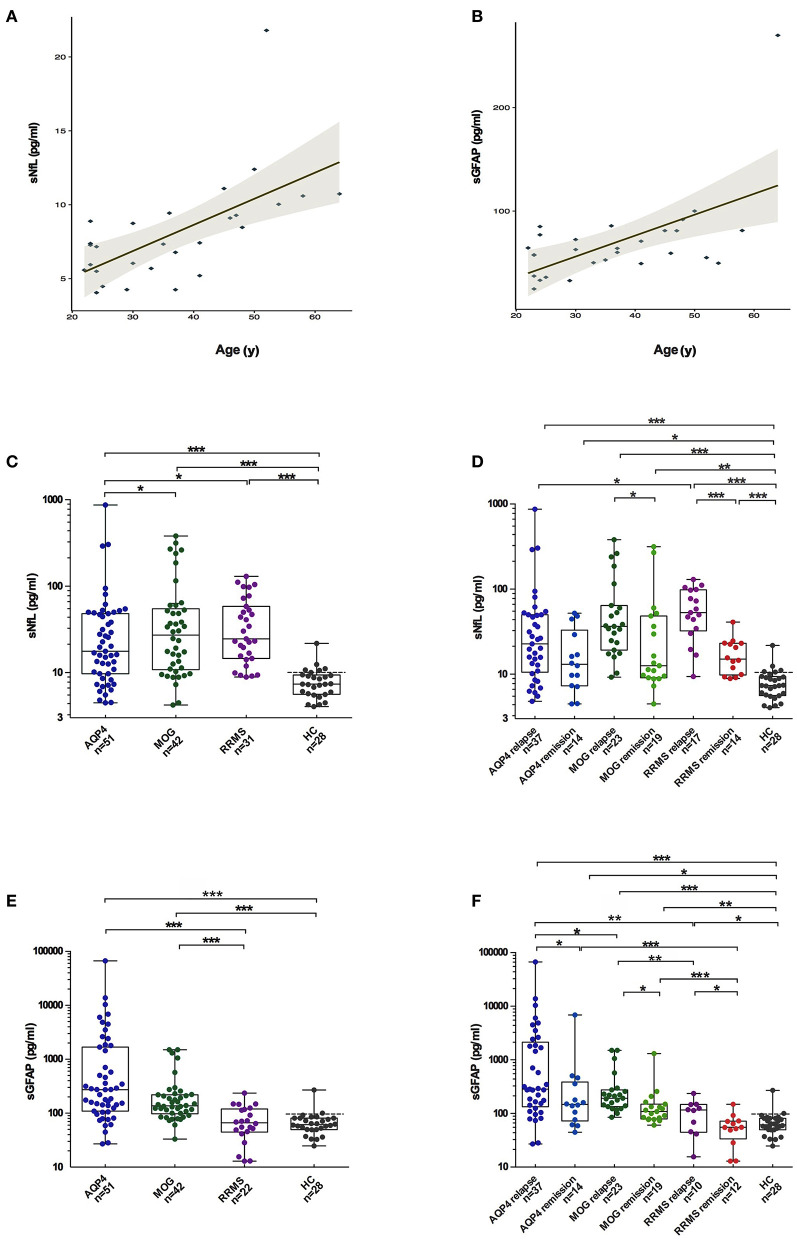
Comparisons of sGFAP and sNfL levels among the different groups. **(A,B)** Correlations of sNfL and sGFAP levels with age in HCs. **(C,E)** sNfL and sGFAP levels were compared among all the samples from patients with AQP4-antibody-positive NMOSD, MOGAD, and RRMS, as well as from HCs. **(D,F)** Samples from the three disease groups were divided into relapse and remission stages and were compared. Boxes depict the median and interquartile range (IQR), with the median represented by the line in the center, and whiskers extending from minimum to maximum values. The dotted lines represent the 90th percentiles of sNfL or sGFAP concentrations in HCs. sNfL, serum neurofilament light; sGFAP, serum glial fibrillary acidic protein; HC, healthy control; AQP4, aquaporin 4; NMOSD, neuromyelitis optica spectrum disorders; MOGAD, myelin oligodendrocyte glycoprotein-antibody-associated diseases; RRMS, relapsing-remitting multiple sclerosis. The *p*-values were obtained with a mixed-effect model adjusted for age and EDSS scores (*p < 0.05, **p < 0.01, and ***p < 0.001). In the figure, AQP4 stands for AQP4-antibody-positive NMOSD; MOG stands for MOGAD.

In HCs, the median sGFAP and sNfL concentrations were 61.4 pg/mL (IQR 49.7–81.0 pg/mL) and 7.4 pg/mL (IQR 5.6–9.4 pg/mL), respectively. Both sGFAP and sNfL levels correlated positively with age (β = 2.247, *p* < 0.001; β = 1.905, *p* = 0.001, respectively), but did not differ between sexes (Figures 1A,B).

### sNfL Levels in Different Disease Groups

The sNfL levels of patients with AQP4-ab+NMOSD [17.6 pg/mL (IQR 9.6–48.1 pg/mL), *p* < 0.001], MOGAD [27.2 pg/mL (IQR 10.8–54.8 pg/Ml), *p* < 0.001], and RRMS [24.5 pg/mL (IQR 14.5–58.3 pg/Ml), *p* < 0.001] were significantly higher compared to those of HCs. Moreover, sNfL levels in patients with MOGAD and RRMS were higher than in patients with AQP4-ab+NMOSD (*p* = 0.023, *p* = 0.027, respectively). No significant difference in sNfL levels was detected between MOGAD and RRMS patients (Figure 1C).

The sNfL levels during relapse were significantly increased compared to those during the remission stage in patients with MOGAD and RRMS [MOGAD: relapse = 34.1 pg/mL (IQR 17.6–64.3 pg/mL), remission = 12.5 pg/mL (IQR 9.1–48.4 pg/mL), *p* = 0.049; RRMS: relapse = 53.1 pg/mL (IQR 32.3–98.2 pg/mL), remission = 15.0 pg/mL (IQR 9.8–23.1 pg/mL), *p* < 0.001]. In contrast, in AQP4-ab+NMOSD patients, sNfL levels during relapse were not significantly different from those during remission [relapse = 22.6 pg/mL (IQR 10.5–50.1 pg/mL), remission = 13.0 pg/mL (IQR 7.6–40.7 pg/mL), *p* = 0.090] (Figure 1D).

The 90th percentile of sNfL levels across the three disease groups was 108.1 pg/mL. Twelve patients exhibited sNfL levels above this value, including three patients with AQP4-ab+NMOSDs (868.2, 304.4, and 291.7 pg/mL), seven patients with MOGADs (381.5, 315.4, 269.7, 262.2, 239.1, 186.1, and 115.8 pg/mL) and two patients with RRMS (130.1 and 111.6 pg/mL).

After removing patients with only optic neuritis (ON) during a recent relapse, the sNfL levels were slightly increased in the AQP4-ab+NMOSD and MOGAD groups, and the difference of sNfL levels between AQP4-ab+NMOSD and RRMS groups diminished (*p* = 0.133) (Supplementary Figure 1).

### sGFAP Levels in Different Disease Groups

The sGFAP levels in the AQP4-ab+NMOSD group [274.1 pg/mL (IQR 109.2–1680.6 pg/mL)] were significantly higher compared to those in the RRMS group [66.5 pg/mL (IQR 44.5–119.7 pg/mL), *p* < 0.001] and those of HCs (*p* < 0.001), and tended to be higher compared to those in the MOGAD group [136.7 pg/mL (IQR 97.8–220.1 pg/mL), *p* = 0.072]. The sGFAP levels were also significantly higher in the MOGAD group compared to those in RRMS and HC groups (*p* < 0.001, *p* < 0.001, respectively) (Figure 1E).

In all three of the disease groups, sGFAP levels were significantly higher during relapse than during remission [AQP4-ab+NMOSD: relapse = 284.4 pg/mL (IQR 133.8–2,144.4 pg/mL), remission = 147.1 pg/mL (IQR 65.0–436.7 pg/mL), *p* = 0.033; MOGAD: relapse = 187.7 pg/mL (IQR 123.3–274.9 pg/mL), remission = 108.5 pg/mL (IQR 77.6–149.4 pg/mL), *p* = 0.015; RRMS: relapse = 117.9 pg/mL (IQR 57.0–147.5 pg/mL), remission = 55.0 pg/mL (IQR 21.9–71.6 pg/mL), *p* = 0.013] (Figure 1F).

The 90th percentile of sGFAP levels was 1,822 pg/mL across the three disease groups. Notably, extremely high levels of sGFAP above the 90th percentile were exclusively seen in patients with AQP4-ab+NMOSD, ranging from 1,865.4 to 67,038.8 pg/mL.

After removing patients with only ON in the AQP4-ab+NMOSD and MOGAD groups, the difference between MOGAD and RRMS group became insignificant. However, the sGFAP concentrations in the AQP4-ab+NMOSD group were still extraordinarily high (Supplementary Figure 1).

### sGFAP/sNfL Ratios

**Figure 2:**
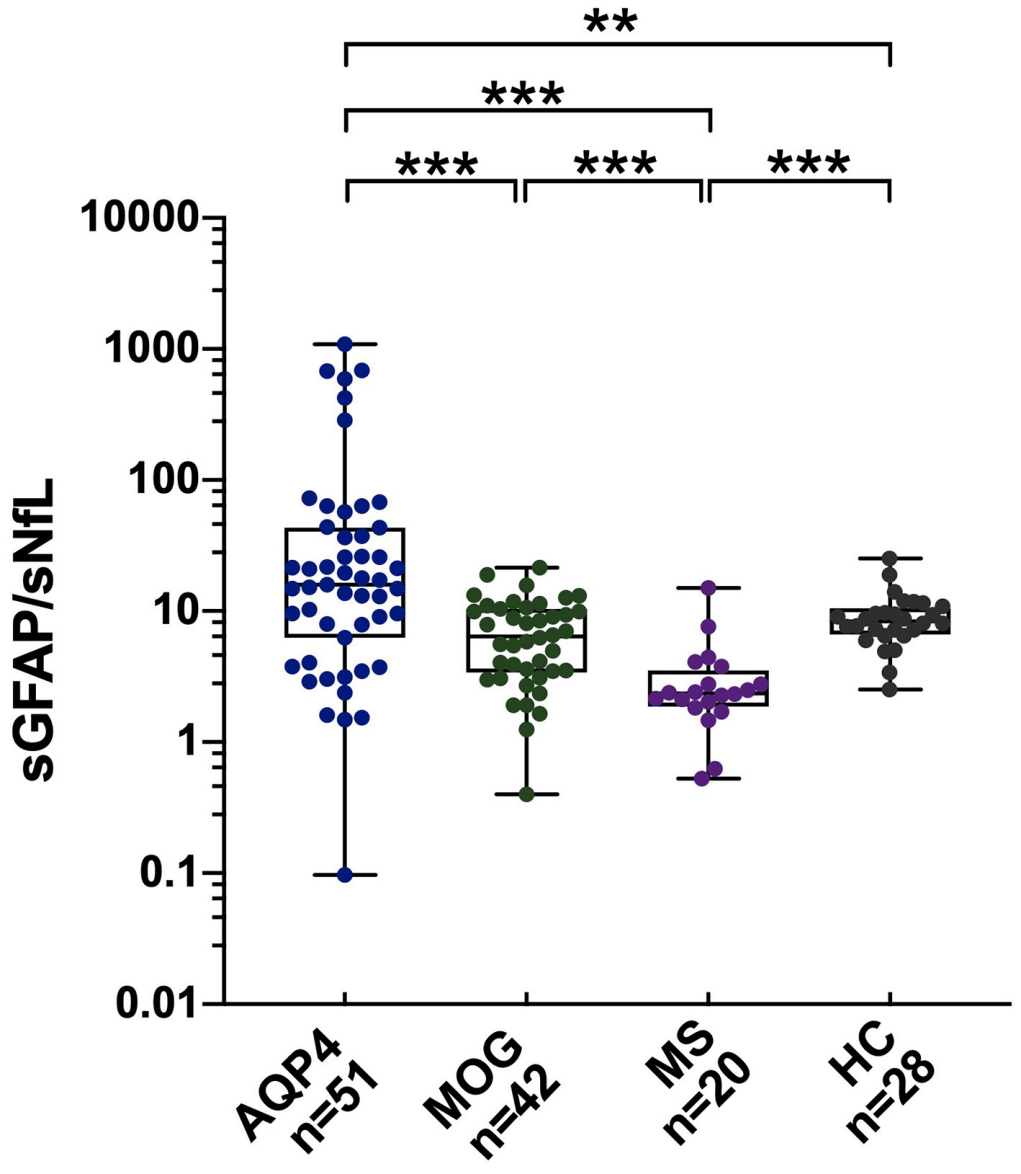
sGFAP/sNfL ratios among the different groups. The sGFAP/sNfL ratios were compared among all the samples from patients with AQP4-antibody-positive NMOSD, MOGAD, and RRMS, as well as from HCs. Boxes depict the median and interquartile range (IQR), with the median represented by the line in the center, and whiskers extending from minimum to maximum values. sNfL, serum neurofilament light; sGFAP, serum glial fibrillary acidic protein; HC, healthy control; AQP4, aquaporin 4; NMOSD, neuromyelitis optica spectrum disorders; MOGAD, myelin oligodendrocyte glycoprotein-antibody-associated diseases; RRMS, relapsing-remitting multiple sclerosis. **p < 0.01, and ***p < 0.001. In the figure, AQP4 stands for AQP4-antibody-positive NMOSD; MOG stands for MOGAD.

The sGFAP/sNfL ratios in the AQP4-ab+NMOSD group [15.8 (IQR 7.1–40.6)] were significantly higher compared to those in the MOGAD group [6.4 (IQR 3.5–10.3), *p* < 0.001], RRMS group [2.3 (IQR 2.0–3.0), *p* < 0.001], and HCs [8.3 (IQR 7.0–10.0), *p* < 0.001]. The sGFAP/sNfL ratios were also higher in the MOGAD group than in the RRMS group (*p* < 0.001) (Figure 2). The multi-comparison results among the three disease groups were similar after excluding patients with only ON (Supplementary Figure 1).

### Association of sNfL and sGFAP Levels With Clinical Parameters in AQP4-ab+ NMOSD

In the AQP4-ab+NMOSD group, both univariate and multivariate models revealed that sNfL levels were positively correlated with EDSS scores (univariate: β = 1.87, *p* = 0.016; multivariate: β = 1.88, *p* = 0.018) (Table 2).

**Table 2:** Clinical relevance of sNfL and sGFAP in AQP4-antibody-positive NMOSD examined by univariate and multivariate regression models.

**Clinical parameter (Number of samples)**	**sNfL (pg/mL)**	**sGFAP (pg/mL)**	**Univariate**_**(sNfL)**_			**Multivariate**_**(sNfL)**_			**Univariate**_**(sGFAP)**_			**Multivariate**_**(sGFAP)**_		
	**Median (IQR)**	**Median (IQR)**	**β**	**95%CI**	***p*-value**	**β**	**95%CI**	***p*-value**	**β**	**95%CI**	***p*-value**	**β**	**95%CI**	***p*-value**
Age at sampling (51)	–	–	1.79	1.75–1.82	0.551	1.17	0.90–1.49	0.612	1.52	1.46–1.58	0.561	1.00	0.68–1.46	0.867
Sex														
Female (44)	16.0 (8.6–48.1)	247.8 (109.2–1793.2)	–		–	–		–	–		–	–		–
Male (7)	46.9 (26.9–51.1)	315.4 (119.3–605.0)	1.83	0.67–4.95	0.256	–		–	1.52	0.33–4.48	0.615	–		–
EDSS score at sampling (51)	–	–	1.87	1.52–2.27	**0.016**	1.88	1.49–2.22	**0.018**	2.45	1.69–3.67	**0.021**	2.04	1.53–2.69	**0.032**
Disease duration (51)	–	–	0.96	0.95–0.96	0.486	–		–	0.84	0.74–0.99	0.542	–		–
Recent relapse (<60 d)														
Yes (37)	22.6 (10.5–50.1)	284.4 (133.8–2144.4)	–		–	–		–	–		–	–		–
No (14)	13.0 (7.6–40.7)	147.1 (65.0–436.7)	1.81	0.82–3.32	0.090	–		–	2.76	2.69–3.00	0.064	–		–
Spinal cord lesion in MRI during recent relapse														
Yes (35)	26.0 (13.3–50.0)	292.8 (145.5–2284.0)	–		–	–		–	–		–	–		–
No (16)	11.5 (6.4–28.8)	149.6 (65.7–338.4)	1.65	0.86–3.16	0.138	–		–	3.29	2.11–5.16	**0.022**	5.30	4.95–6.43	**0.008**
Brain lesion in MRI during recent relapse														
Yes (16)	27.1 (20.2–27.7)	388.9 (179.2–2523.6)	–		–	–		–	–		–	–		–
No (35)	13.4 (7.3–49.6)	159.6 (100.1–540.9)	1.75	1.49–2.01	0.094	–		–	2.27	0.83–5.47	0.121	–		–
Treatment at sampling														
Treated (38)	21.2 (8.8–51.4)	203.7 (128.2–466.4)	–		–	–		–	–		–	–		–
No treatment (13)	15.8 (10.4–33.5)	579.9 (97.9–4769.5)	1.53	0.74–3.00	0.238	–		–	1.89	0.65–4.44	0.261	–		–

*sNfL, serum neurofilament light; sGFAP, serum glial fibrillary acidic protein; AQP4, aquaporin 4; NMOSD, neuromyelitis optica spectrum disorders; IQR, interquartile range; CI: confidence interval; EDSS, Expanded Disability Status Scale; MRI, magnetic resonance imaging. Estimates described in this table used the original scales (β). Treated means usage of steroid and/or immunosuppressant (includes oral immunosuppressant and rituximab). The p-values lower than 0.05 are in bold type*.

As for sGFAP, both univariate and multivariate analysis revealed that sGFAP levels correlated positively with EDSS scores (univariate: β = 2.45, *p* = 0.021; multivariate: β = 2.04, *p* = 0.032), and were higher in those with spinal cord lesions during a recent relapse (univariate: β = 3.29, *p* = 0.022; multivariate: β = 5.30, *p* = 0.008) (Table 2).

### Association of sNfL and sGFAP Levels With Clinical Parameters in MOGAD

Within the MOGAD group, univariate analysis showed that sNfL levels were significantly higher in male patients than in female patients (β = 2.60, *p* = 0.007), but this was not further validated in multivariate analysis. Both univariate and multivariate analyses showed that sNfL levels were significantly higher in patients with brain lesions on magnetic resonance imaging (MRI) during a recent attack (β = 3.03, *p* = 0.002; β = 2.80, *p* = 0.023, respectively), and revealed a positive correlation between sNfL levels and EDSS scores (β = 1.52, *p* = 0.013; β = 1.98, *p* = 0.013, respectively) (Table 3).

**Table 3:** Clinical relevance of sNfL and sGFAP in MOGAD examined by univariate and multivariate regression models.

**Clinical parameter (Number of samples)**	**sNfL (pg/mL)**	**sGFAP (pg/mL)**	**Univariate**_**(sNfL)**_			**Multivariate**_**(sNfL)**_			**Univariate**_**(sGFAP)**_			**Multivariate**_**(sGFAP)**_		
	**Median (IQR)**	**Median (IQR)**	**β**	**95%CI**	***p*-value**	**β**	**95%CI**	***p*-value**	**β**	**95%CI**	***p*-value**	**β**	**95%CI**	***p*-value**
Age at sampling (42)	–	–	1.28	1.01–1.49	0.933	0.98	0.97–1.11	0.386	1.50	1.47–1.52	0.274	1.38	1.36–1.39	**0.048**
Sex														
Female (22)	11.2 (9.0–26.0)	116.6 (84.1–171.2)	–		–	–		–	–		–	–		–
Male (20)	43.4 (31.3–63.4)	182.5 (125.2–270.4)	2.60	1.31–4.90	**0.007**	1.13	0.54–2.27	0.422	1.51	0.90–2.36	0.118	–		–
EDSS score at sampling (42)	–	–	1.52	1.23–1.85	**0.013**	1.98	1.49–2.41	**0.013**	1.74	1.49–2.01	**0.010**	1.52	1.36–1.66	**0.008**
Disease duration (42)	–	–	0.98	0.97–0.99	0.597	–		–	0.94	0.94–0.95	0.221	–		–
Recent relapse (<60 d)														
Yes (23)	34.1 (17.6–64.3)	187.7 (123.3–274.9)	–		–	–		–	–		–	–		–
No (19)	12.5 (9.1–48.4)	108.5 (77.6–149.4)	2.05	1.01–4.54	**0.049**	1.71	0.90–3.00	0.205	1.86	1.14–3.00	**0.015**	1.61	1.01–2.23	0.061
Spinal cord lesion in MRI during recent relapse														
Yes (14)	26.4 (10.8–244.9)	141.9 (93.8–217.2)	–		–	–		–	–		–	–		–
No (28)	27.1 (10.4–48.4)	131.8 (101.1–456.7)	1.57	1.24–1.82	0.253	–		–	1.34	0.74–2.01	0.298	–		–
Brain lesion in MRI during recent relapse														
Yes (22)	42.7 (25.5–133.4)	189.8 (106.5–280.9)	–		–	–		–	–		–	–		–
No (20)	11.9 (9.1–24.4)	122.8 (84.7–146.7)	3.03	1.42–5.44	**0.002**	2.80	1.20–4.32	**0.023**	1.71	1.00–2.71	**0.036**	1.61	1.02–2.45	**0.015**
Treatment at sampling														
Treated (37)	19.2 (9.9–50.2)	133.1 (88.4–220.9)	–		–	–		–	–		–	–		–
No treatment (5)	64.3(36.6–188.9)	144.9(131.3–623.9)	2.87	1.25–4.91	0.061	–		–	1.36	0.74–2.46	0.452	–		–

*sNfL, serum neurofilament light; sGFAP, serum glial fibrillary acidic protein; MOGAD, myelin oligodendrocyte glycoprotein-antibody-associated disease; IQR, interquartile range; CI: confidence interval; EDSS, Expanded Disability Status Scale; MRI, magnetic resonance imaging. Estimates described in this table used the original scales (β). Treated means usage of steroid and/or immunosuppressant (includes oral immunosuppressant and rituximab). The p-values lower than 0.05 are in bold type*.

Univariate and multivariate analyses also indicated that sGFAP levels correlated positively with EDSS scores (β = 1.74, *p* = 0.010; β = 1.52, *p* = 0.008, respectively) and were higher in patients with brain lesions during a recent relapse (β = 1.71, *p* = 0.036; β = 1.61, *p* = 0.015, respectively), whereas univariate but not multivariate analysis revealed significantly higher sGFAP levels in MOGAD patients who experienced a relapse within 60 days (β = 1.86, *p* = 0.015) (Table 3).

## Discussion

The main findings of the present investigation were as follows: (1) sNfL levels were increased significantly in patients with AQP4-ab-positive NMOSD, MOGAD, and RRMS compared to those in HCs; (2) sGFAP levels were remarkably higher in AQP4-ab-positive NMOSD and MOGAD patients than in HCs; (3) The sGFAP/sNfL ratio exhibited good discrimination among the three disease groups; (4) sNfL levels were increased during relapse in patients with MOGAD and RRMS, while sGFAP levels were increased during relapse in all three of the disease groups; and (5) Both sNfL and sGFAP concentrations correlated with EDSS scores in AQP4-ab-positive NMOSD and MOGAD patients.

Elevation of sNfL levels is a reflection of underlying neuronal-axonal damage, which is not only seen in MS, but also in AQP4- and MOG-ab-associated inflammatory diseases (27–31). In NMOSD, possible reasons for neuronal-axonal damage may be secondary anterograde/retrograde degeneration, loss of trophic support following the destruction of astrocytes, mitochondrial damage, and axonal energy failure (35). Interestingly, unlike MS, we did not observe a significant change in sNfL levels between relapse and remission phases in NMOSD patients, similar to the observation of Watanabe et al. (30). This suggests that neuronal damage in NMOSD may not be directly associated with acute inflammation targeting astrocytes, but may instead indicate a sustained secondary pathological process. In MOGAD, the primary pathological damage occurs in myelin and oligodendrocytes. Predominant demyelination has been observed in brain lesions of MOGAD patients; additionally, CSF myelin basic protein, a marker for myelin injury, is significantly increased in MOGAD (31). Therefore, the elevation of sNfL levels in MOGAD may be explained by the successive impairment of axons following myelin damage.

Among the three disease groups in the present study, the sGFAP levels were the highest in patients with AQP4-ab+NMOSD. Furthermore, extremely high sGFAP concentrations (> 90th percentile) were exclusively seen in patients with AQP4-ab+NMOSD. These results are in accordance with our expectations and a previous observation by Watanabe et al. (30), as GFAP is abundantly expressed in astrocytes, which are the primary targets of autoreactive AQP4-ab (2, 3). In a previous study, GFAP levels were also found to be higher in the CSF of AQP4-ab+NMOSD patients, compared with those in the CSF of MOGAD and MS patients, although the investigators used enzyme-linked immunosorbent assays rather than SIMOA to measure GFAP concentrations (31). The elevation of sGFAP levels in MOGAD patients may be explained by secondary astrocytic damage during oligodendrocyte-targeted inflammation, or by enhanced GFAP expression caused by astrocytic activation or reactive astrogliosis. Previous researchers have also demonstrated increased GFAP in MS, especially in progressive MS (25, 26). However, we did not observe increased levels of sGFAP in patients with RRMS, although astrocytic activation or reactive gliosis can also be expected in MS. A possible explanation is that we only included the relapsing-remitting type of MS with relatively lower EDSS scores, whereas elevated sGFAP might be more prominent in patients at a progressive stage.

Different sNfL and sGFAP levels represent different weights of astrocytopathy and neuronal-axonal injury within one disease. In the present study, AQP4-ab-positive NMOSD patients exhibited the highest sGFAP/sNfL ratios, indicating its dominant astrocytopathic nature; RRMS was at the other extreme, such that neuronal-axonal impairment prevailed over astrocytopathy; and MOGAD was in the middle of them (Figure 2, Supplementary Figure 1). This concept has also been proposed in a previous investigation by Watanabe et al. (30), in which the sGFAP/sNfL ratio was first reported to be higher in NMOSD than in MS patients, especially during NMOSD relapse, although their study included both AQP4-ab-positive and -negative NMOSD patients. Our present study further supports the viewpoint that the sGFAP/sNfL ratio reflects distinct disease pathophysiology between NMOSD and MS, and we provided further information in regard to MOGAD on this issue.

We also noticed that within the AQP4-ab+NMOSD group, sGFAP levels were higher in patients with spinal cord lesions during a recent relapse than in those without spinal cord lesions; and within the MOGAD group, sGFAP and sNfL levels were higher in patients with recent brain lesions. We speculate that lesion volumes may possibly affect the concentrations of the biomarkers, as lesions in the spinal cord or brain are usually much larger than those in the optic nerves. For this reason, we also performed multi-comparisons excluding patients with only ON. The comparisons of individual biomarkers showed mild difference mainly because of the slight elevation of biomarker concentrations in the AQP4+NMOSD and MOGAD groups. However, the comparison of sGFAP/sNfL ratios among the three disease groups exhibited the same trend irrespective of excluding or including patients with only ON, indicating that the ratio is more efficient in reflecting different disease pathogenesis and less affected by lesion volume.

In the current study, sNfL and sGFAP levels measured by ultra-sensitive SIMOA assays showed significant positive correlations with EDSS scores in both AQP4-ab+NMOSD and MOGAD groups and tended to increase during acute relapses, indicating that they are potential biomarkers for measuring disease severity and activity.

Our present study had limitations due to its cross-sectional nature. Associations between clinical parameters and sNfL or sGFAP levels were not analyzed in relapse/remission subgroups or in RRMS group due to the limited sample sizes. Besides, the impacts of steroid or immunosuppressants on the concentrations of biomarkers were not fully elaborated in the current study. Thus, all results should be conservatively interpreted and only have an exploratory character and subsequently validated in a larger prospective cohort.

## Conclusion

In conclusion, we provided further supportive evidence that sNfL and sGFAP levels were associated with disease severity in AQP4-ab+NMOSD and MOGAD, and that sGFAP/sNfL ratios reflected distinct disease pathophysiology of CNS inflammatory disorders.

## Data Availability Statement

The original contributions presented in the study are included in the article/Supplementary Material, further inquiries can be directed to the corresponding author/s.

## Ethics Statement

The studies involving human participants were reviewed and approved by the Medical Ethics Committee of Huashan Hospital Fudan University and have therefore been performed in accordance with the ethical standards laid down in the 1964 Declaration of Helsinki and its later amendments. The patients/participants provided their written informed consent to participate in this study.

## Author Contributions

All authors listed have made a substantial, direct and intellectual contribution to the work, and approved it for publication.

## Conflict of Interest

The authors declare that the research was conducted in the absence of any commercial or financial relationships that could be construed as a potential conflict of interest.

## Supplementary Material

Supplementary Figure 1 sGFAP, sNfL levels, and sGFAP/sNfL ratios among the different groups excluding patients with only ON. sNfL **(A)**, sGFAP levels **(B)** and sGFAP/sNfL ratios **(C)** were compared among patients with AQP4-antibody-positive NMOSD, MOGAD, and RRMS, as well as HCs. Patients with only ON during a recent relapse in AQP4-antibody-positive NMOSD and MOGAD group were excluded from the analyses. Boxes depict the median and interquartile range (IQR), with the median represented by the line in the center, and whiskers extending from minimum to maximum values. The dotted lines represent the 90th percentiles of sNfL or sGFAP concentrations in HCs. sGFAP, serum glial fibrillary acidic protein; sNfL, serum neurofilament light; ON, optic neuritis; AQP4, aquaporin 4; NMOSD, neuromyelitis optica spectrum disorders; MOGAD, myelin oligodendrocyte glycoprotein-antibody-associated diseases; RRMS, relapsing-remitting multiple sclerosis; HC, healthy controls. The *p*-values were obtained with a mixed-effect model adjusted for age and EDSS scores (^*^*p* < 0.05, ^**^*p* < 0.01, and ^***^*p* < 0.001). In the figure, AQP4 stands for AQP4-antibody-positive NMOSD; MOG stands for MOGAD.
