# Supplementary figures and images for: Serum Neurofilament Light and GFAP Are Associated With Disease Severity in Inflammatory Disorders With Aquaporin-4 or Myelin Oligodendrocyte Glycoprotein Antibodies
Source: Front Immunol. 2021 Mar 16;12:647618. doi: 10.3389/fimmu.2021.647618 (PMC8008082; doi:10.3389/fimmu.2021.647618)

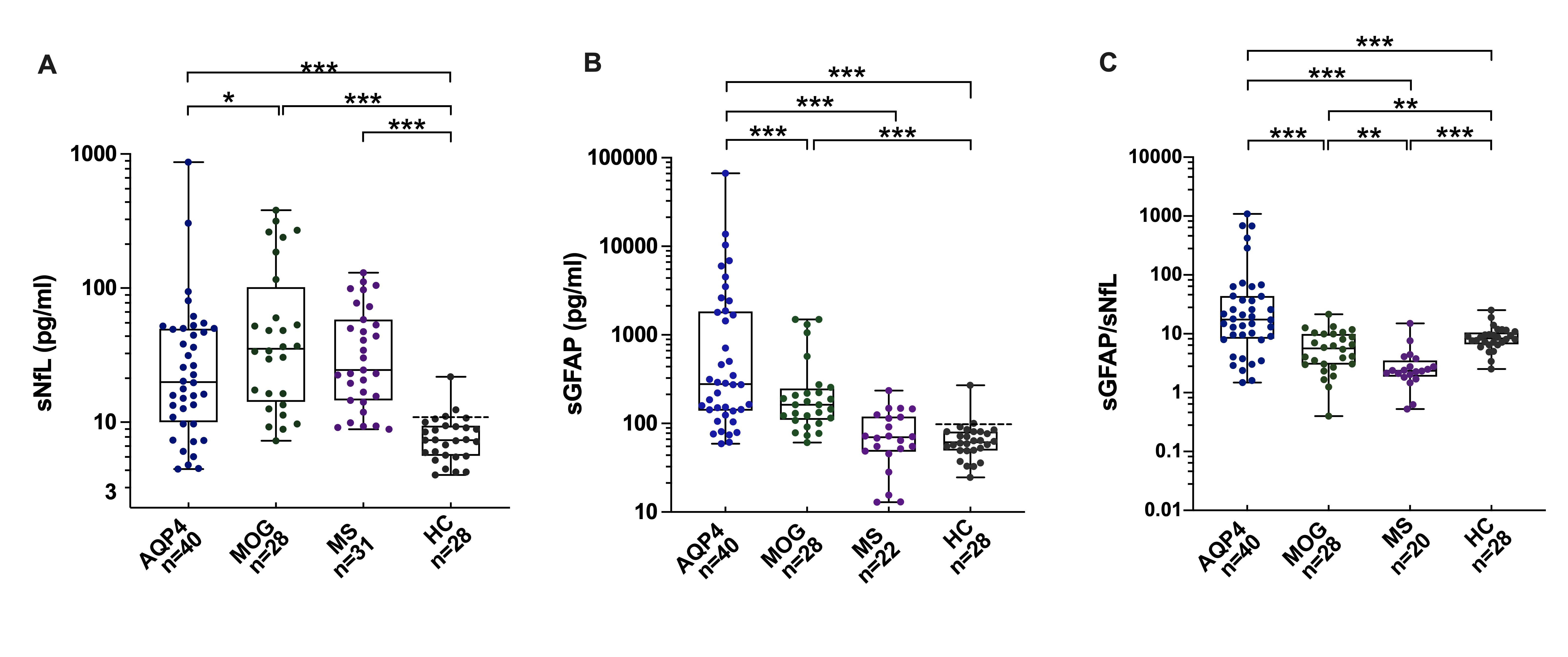

Supplement: Supplementary Figure 1 — sGFAP, sNfL levels, and sGFAP/sNfL ratios among the different groups excluding patients with only ON. sNfL (A), sGFAP levels (B) and sGFAP/sNfL ratios (C) were compared among patients with AQP4-antibody-positive NMOSD, MOGAD, and RRMS, as well as HCs. Patients with only ON during a recent relapse in AQP4-antibody-positive NMOSD and MOGAD group were excluded from the analyses. Boxes depict the median and interquartile range (IQR), with the median represented by the line in the center, and whiskers extending from minimum to maximum values. The dotted lines represent the 90th percentiles of sNfL or sGFAP concentrations in HCs. sGFAP, serum glial fibrillary acidic protein; sNfL, serum neurofilament light; ON, optic neuritis; AQP4, aquaporin 4; NMOSD, neuromyelitis optica spectrum disorders; MOGAD, myelin oligodendrocyte glycoprotein-antibody-associated diseases; RRMS, relapsing-remitting multiple sclerosis; HC, healthy controls. The p-values were obtained with a mixed-effect model adjusted for age and EDSS scores (*p < 0.05, **p < 0.01, and ***p < 0.001). In the figure, AQP4 stands for AQP4-antibody-positive NMOSD; MOG stands for MOGAD. [file Image_1.JPEG]
